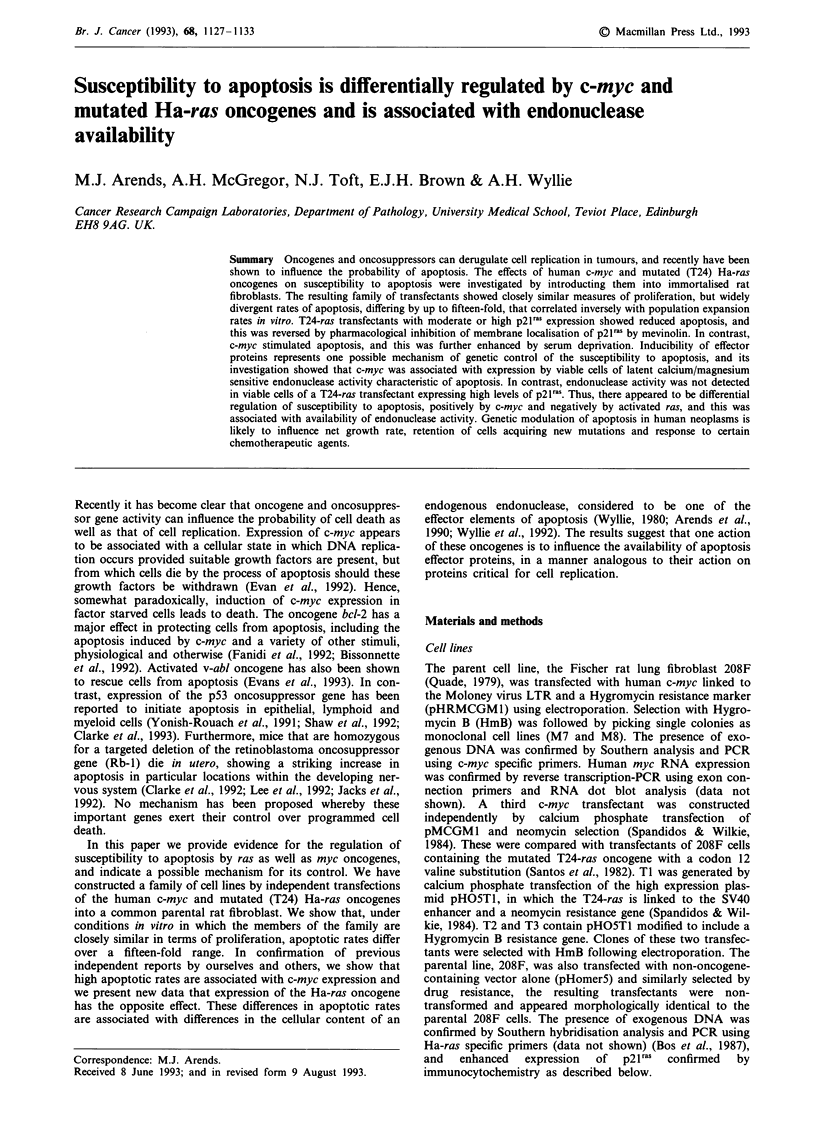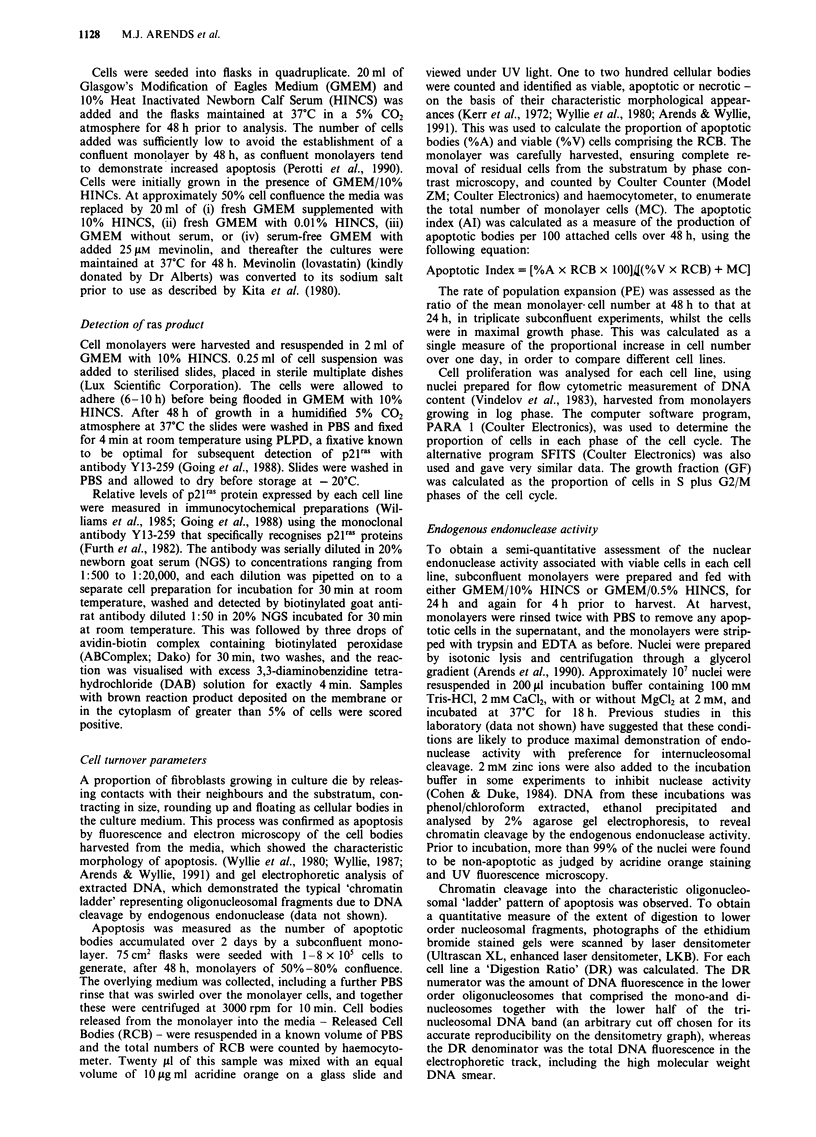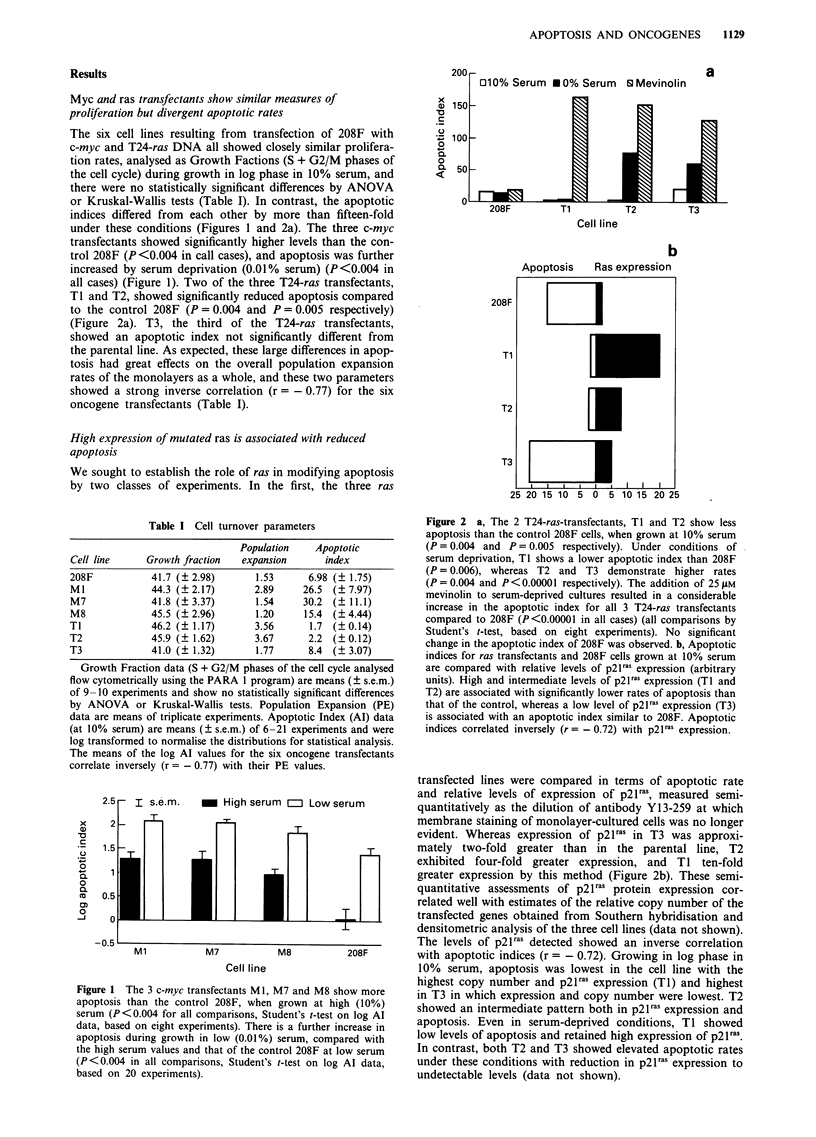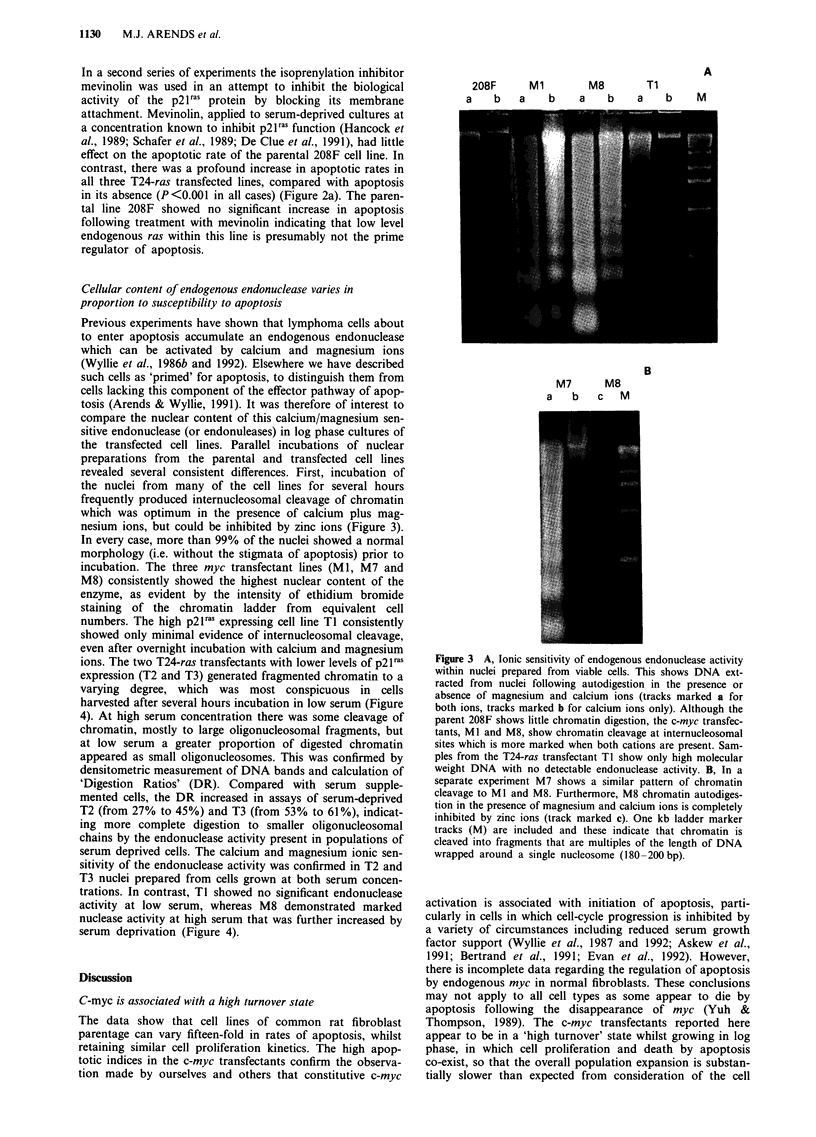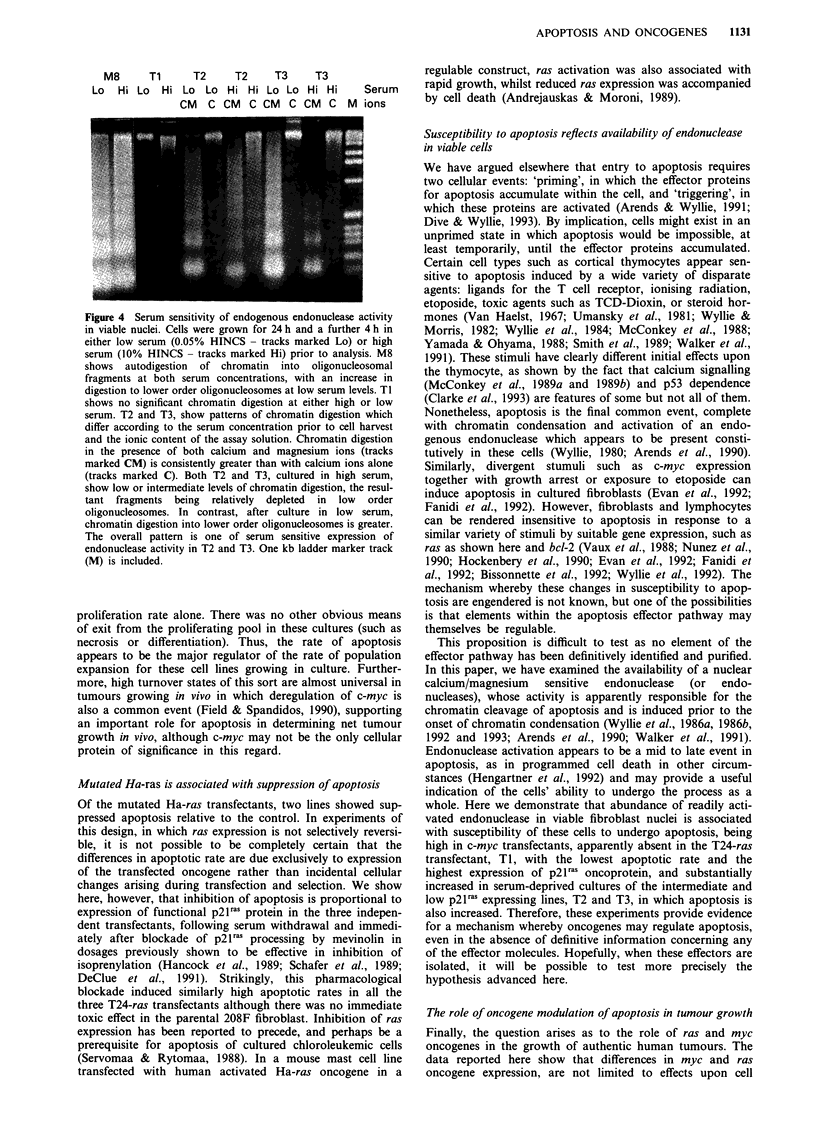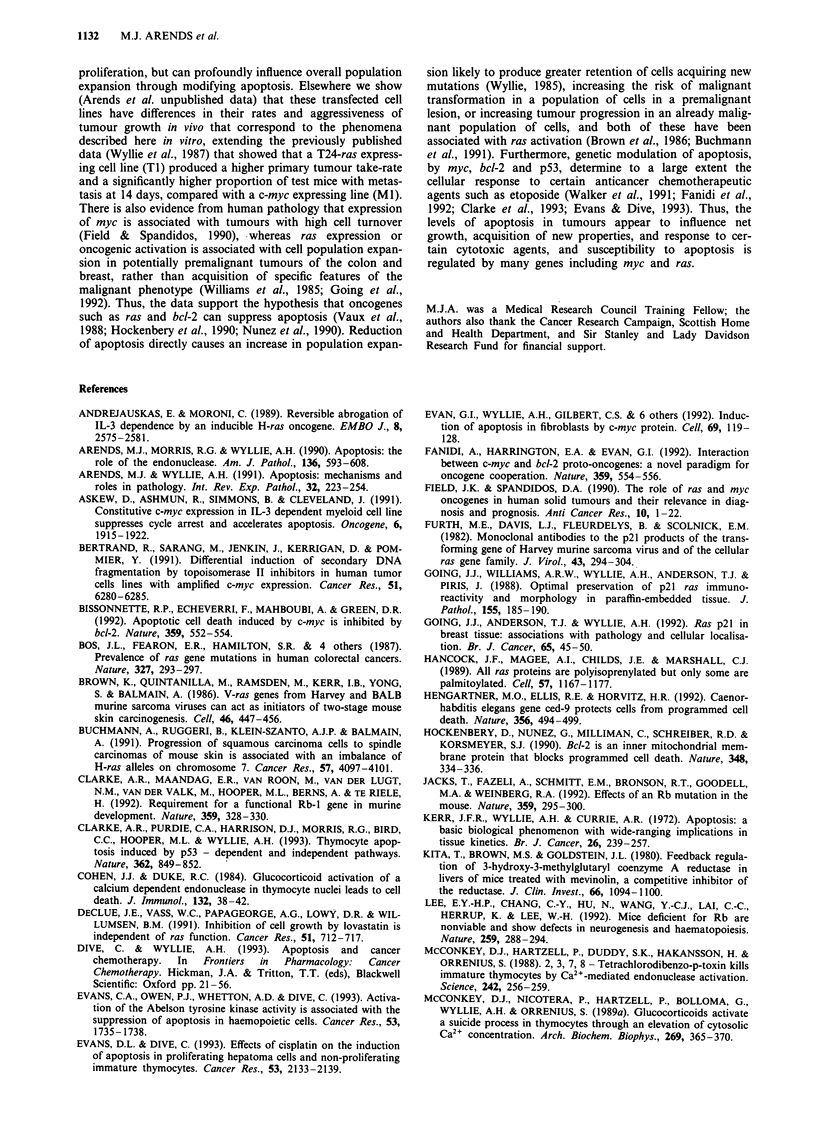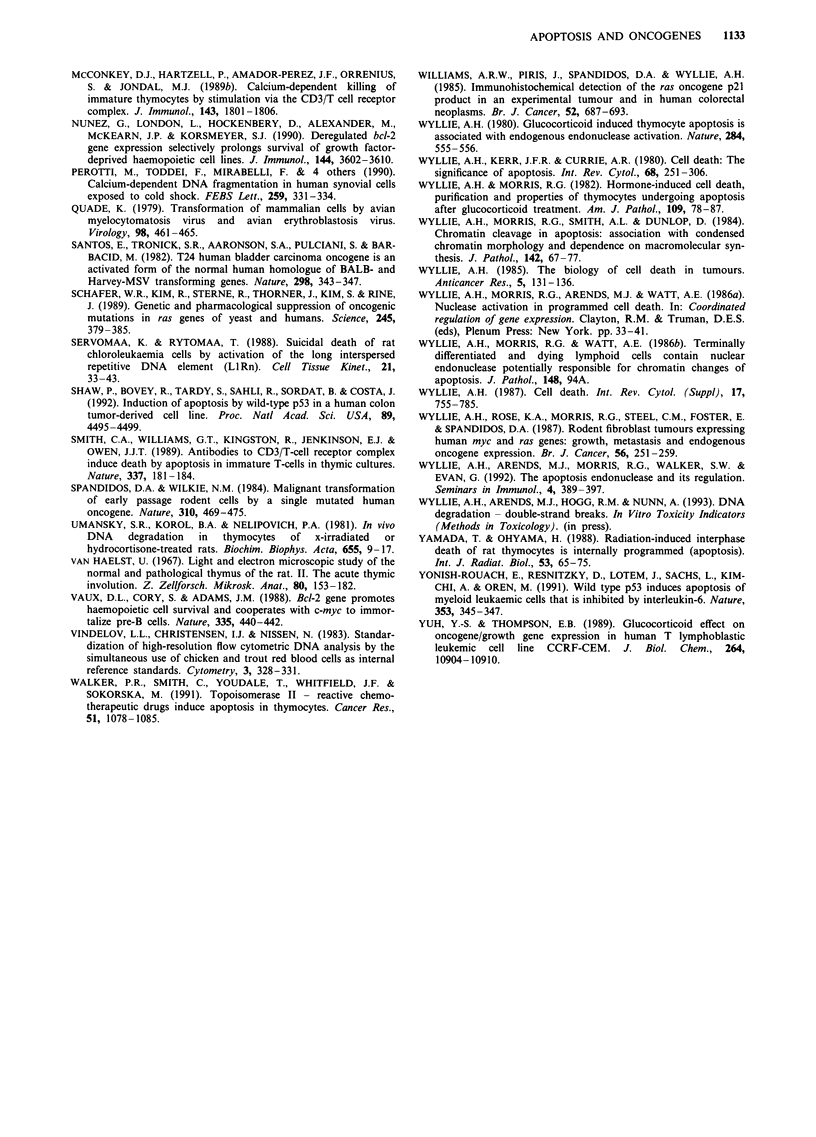# Susceptibility to apoptosis is differentially regulated by c-myc and mutated Ha-ras oncogenes and is associated with endonuclease availability.

**DOI:** 10.1038/bjc.1993.492

**Published:** 1993-12

**Authors:** M. J. Arends, A. H. McGregor, N. J. Toft, E. J. Brown, A. H. Wyllie

**Affiliations:** Department of Pathology, University Medical School, Edinburgh, UK.

## Abstract

**Images:**


*Br. J. Cancer* (1993), **68**, 1127–1133
© Macmillan Press Ltd., 1993

### Susceptibility to apoptosis is differentially regulated by c-*myc* and mutated Ha-*ras* oncogenes and is associated with endonuclease availability

M.J. Arends, A.H. McGregor, N.J. Toft, E.J.H. Brown & A.H. Wyllie

*Cancer Research Campaign Laboratories, Department of Pathology, University Medical School, Teviot Place, Edinburgh EH8 9AG. UK.*

**Summary** Oncogenes and oncosuppressors can derugulate cell replication in tumours, and recently have been shown to influence the probability of apoptosis. The effects of human c-*myc* and mutated (T24) Ha-*ras* oncogenes on susceptibility to apoptosis were investigated by introducting them into immortalised rat fibroblasts. The resulting family of transfectants showed closely similar measures of proliferation, but widely divergent rates of apoptosis, differing by up to fifteen-fold, that correlated inversely with population expansion rates *in vitro*. T24-*ras* transfectants with moderate or high $p21^{ras}$ expression showed reduced apoptosis, and this was reversed by pharmacological inhibition of membrane localisation of $p21^{ras}$ by mevinolin. In contrast, c-*myc* stimulated apoptosis, and this was further enhanced by serum deprivation. Inducibility of effector proteins represents one possible mechanism of genetic control of the susceptibility to apoptosis, and its investigation showed that c-*myc* was associated with expression by viable cells of latent calcium/magnesium sensitive endonuclease activity characteristic of apoptosis. In contrast, endonuclease activity was not detected in viable cells of a T24-*ras* transfectant expressing high levels of $p21^{ras}$. Thus, there appeared to be differential regulation of susceptibility to apoptosis, positively by c-*myc* and negatively by activated *ras*, and this was associated with availability of endonuclease activity. Genetic modulation of apoptosis in human neoplasms is likely to influence net growth rate, retention of cells acquiring new mutations and response to certain chemotherapeutic agents.

Recently it has become clear that oncogene and oncosuppressor gene activity can influence the probability of cell death as well as that of cell replication. Expression of c-*myc* appears to be associated with a cellular state in which DNA replication occurs provided suitable growth factors are present, but from which cells die by the process of apoptosis should these growth factors be withdrawn (Evan *et al.*, 1992). Hence, somewhat paradoxically, induction of c-*myc* expression in factor starved cells leads to death. The oncogene *bcl*-2 has a major effect in protecting cells from apoptosis, including the apoptosis induced by c-*myc* and a variety of other stimuli, physiological and otherwise (Fanidi *et al.*, 1992; Bissonnette *et al.*, 1992). Activated v-*abl* oncogene has also been shown to rescue cells from apoptosis (Evans *et al.*, 1993). In contrast, expression of the p53 oncosuppressor gene has been reported to initiate apoptosis in epithelial, lymphoid and myeloid cells (Yonish-Rouach *et al.*, 1991; Shaw *et al.*, 1992; Clarke *et al.*, 1993). Furthermore, mice that are homozygous for a targeted deletion of the retinoblastoma oncosuppressor gene (Rb-1) die *in utero*, showing a striking increase in apoptosis in particular locations within the developing nervous system (Clarke *et al.*, 1992; Lee *et al.*, 1992; Jacks *et al.*, 1992). No mechanism has been proposed whereby these important genes exert their control over programmed cell death.

In this paper we provide evidence for the regulation of susceptibility to apoptosis by *ras* as well as *myc* oncogenes, and indicate a possible mechanism for its control. We have constructed a family of cell lines by independent transfections of the human c-*myc* and mutated (T24) Ha-*ras* oncogenes into a common parental rat fibroblast. We show that, under conditions *in vitro* in which the members of the family are closely similar in terms of proliferation, apoptotic rates differ over a fifteen-fold range. In confirmation of previous independent reports by ourselves and others, we show that high apoptotic rates are associated with c-*myc* expression and we present new data that expression of the Ha-*ras* oncogene has the opposite effect. These differences in apoptotic rates are associated with differences in the cellular content of an endogenous endonuclease, considered to be one of the effector elements of apoptosis (Wyllie, 1980; Arends *et al.*, 1990; Wyllie *et al.*, 1992). The results suggest that one action of these oncogenes is to influence the availability of apoptosis effector proteins, in a manner analogous to their action on proteins critical for cell replication.

Correspondence: M.J. Arends.
Received 8 June 1993; and in revised form 9 August 1993.

### Materials and methods

### *Cell lines*

The parent cell line, the Fischer rat lung fibroblast 208F (Quade, 1979), was transfected with human c-*myc* linked to the Moloney virus LTR and a Hygromycin resistance marker (pHRMCGM1) using electroporation. Selection with Hygromycin B (HmB) was followed by picking single colonies as monoclonal cell lines (M7 and M8). The presence of exogenous DNA was confirmed by Southern analysis and PCR using c-*myc* specific primers. Human *myc* RNA expression was confirmed by reverse transcription-PCR using exon connection primers and RNA dot blot analysis (data not shown). A third c-*myc* transfectant was constructed independently by calcium phosphate transfection of pMCGM1 and neomycin selection (Spandidos & Wilkie, 1984). These were compared with transfectants of 208F cells containing the mutated T24-*ras* oncogene with a codon 12 valine substitution (Santos *et al.*, 1982). T1 was generated by calcium phosphate transfection of the high expression plasmid pHO5T1, in which the T24-*ras* is linked to the SV40 enhancer and a neomycin resistance gene (Spandidos & Wilkie, 1984). T2 and T3 contain pHO5T1 modified to include a Hygromycin B resistance gene. Clones of these two transfectants were selected with HmB following electroporation. The parental line, 208F, was also transfected with non-oncogene-containing vector alone (pHomer5) and similarly selected by drug resistance, the resulting transfectants were non-transformed and appeared morphologically identical to the parental 208F cells. The presence of exogenous DNA was confirmed by Southern hybridisation analysis and PCR using Ha-*ras* specific primers (data not shown) (Bos *et al.*, 1987), and enhanced expression of $p21^{ras}$ confirmed by immunocytochemistry as described below.

Cells were seeded into flasks in quadruplicate. 20 ml of Glasgow's Modification of Eagles Medium (GMEM) and 10% Heat Inactivated Newborn Calf Serum (HINCS) was added and the flasks maintained at 37°C in a 5% $CO_2$ atmosphere for 48 h prior to analysis. The number of cells added was sufficiently low to avoid the establishment of a confluent monolayer by 48 h, as confluent monolayers tend to demonstrate increased apoptosis (Perotti *et al.*, 1990). Cells were initially grown in the presence of GMEM/10% HINCs. At approximately 50% cell confluence the media was replaced by 20 ml of (i) fresh GMEM supplemented with 10% HINCS, (ii) fresh GMEM with 0.01% HINCS, (iii) GMEM without serum, or (iv) serum-free GMEM with added 25 μM mevinolin, and thereafter the cultures were maintained at 37°C for 48 h. Mevinolin (lovastatin) (kindly donated by Dr Alberts) was converted to its sodium salt prior to use as described by Kita *et al.* (1980).

*Detection of* ras *product*

Cell monolayers were harvested and resuspended in 2 ml of GMEM with 10% HINCS. 0.25 ml of cell suspension was added to sterilised slides, placed in sterile multiplate dishes (Lux Scientific Corporation). The cells were allowed to adhere (6–10 h) before being flooded in GMEM with 10% HINCS. After 48 h of growth in a humidified 5% $CO_2$ atmosphere at 37°C the slides were washed in PBS and fixed for 4 min at room temperature using PLPD, a fixative known to be optimal for subsequent detection of $p21^{ras}$ with antibody Y13-259 (Going *et al.*, 1988). Slides were washed in PBS and allowed to dry before storage at − 20°C.

Relative levels of $p21^{ras}$ protein expressed by each cell line were measured in immunocytochemical preparations (Williams *et al.*, 1985; Going *et al.*, 1988) using the monoclonal antibody Y13-259 that specifically recognises $p21^{ras}$ proteins (Furth *et al.*, 1982). The antibody was serially diluted in 20% newborn goat serum (NGS) to concentrations ranging from 1:500 to 1:20,000, and each dilution was pipetted on to a separate cell preparation for incubation for 30 min at room temperature, washed and detected by biotinylated goat anti-rat antibody diluted 1:50 in 20% NGS incubated for 30 min at room temperature. This was followed by three drops of avidin-biotin complex containing biotinylated peroxidase (ABComplex; Dako) for 30 min, two washes, and the reaction was visualised with excess 3,3-diaminobenzidine tetrahydrochloride (DAB) solution for exactly 4 min. Samples with brown reaction product deposited on the membrane or in the cytoplasm of greater than 5% of cells were scored positive.

*Cell turnover parameters*

A proportion of fibroblasts growing in culture die by releasing contacts with their neighbours and the substratum, contracting in size, rounding up and floating as cellular bodies in the culture medium. This process was confirmed as apoptosis by fluorescence and electron microscopy of the cell bodies harvested from the media, which showed the characteristic morphology of apoptosis. (Wyllie *et al.*, 1980; Wyllie, 1987; Arends & Wyllie, 1991) and gel electrophoretic analysis of extracted DNA, which demonstrated the typical 'chromatin ladder' representing oligonucleosomal fragments due to DNA cleavage by endogenous endonuclease (data not shown).

Apoptosis was measured as the number of apoptotic bodies accumulated over 2 days by a subconfluent monolayer. 75 $cm^2$ flasks were seeded with $1-8 \times 10^5$ cells to generate, after 48 h, monolayers of 50%–80% confluence. The overlying medium was collected, including a further PBS rinse that was swirled over the monolayer cells, and together these were centrifuged at 3000 rpm for 10 min. Cell bodies released from the monolayer into the media – Released Cell Bodies (RCB) – were resuspended in a known volume of PBS and the total numbers of RCB were counted by haemocytometer. Twenty μl of this sample was mixed with an equal volume of 10 μg ml acridine orange on a glass slide and viewed under UV light. One to two hundred cellular bodies were counted and identified as viable, apoptotic or necrotic – on the basis of their characteristic morphological appearances (Kerr *et al.*, 1972; Wyllie *et al.*, 1980; Arends & Wyllie, 1991). This was used to calculate the proportion of apoptotic bodies (%A) and viable (%V) cells comprising the RCB. The monolayer was carefully harvested, ensuring complete removal of residual cells from the substratum by phase contrast microscopy, and counted by Coulter Counter (Model ZM; Coulter Electronics) and haemocytometer, to enumerate the total number of monolayer cells (MC). The apoptotic index (AI) was calculated as a measure of the production of apoptotic bodies per 100 attached cells over 48 h, using the following equation:

$$\text{Apoptotic Index} = [\%A \times RCB \times 100]/[(\%V \times RCB) + MC]$$

The rate of population expansion (PE) was assessed as the ratio of the mean monolayer cell number at 48 h to that at 24 h, in triplicate subconfluent experiments, whilst the cells were in maximal growth phase. This was calculated as a single measure of the proportional increase in cell number over one day, in order to compare different cell lines.

Cell proliferation was analysed for each cell line, using nuclei prepared for flow cytometric measurement of DNA content (Vindelov *et al.*, 1983), harvested from monolayers growing in log phase. The computer software program, PARA 1 (Coulter Electronics), was used to determine the proportion of cells in each phase of the cell cycle. The alternative program SFITS (Coulter Electronics) was also used and gave very similar data. The growth fraction (GF) was calculated as the proportion of cells in S plus G2/M phases of the cell cycle.

*Endogenous endonuclease activity*

To obtain a semi-quantitative assessment of the nuclear endonuclease activity associated with viable cells in each cell line, subconfluent monolayers were prepared and fed with either GMEM/10% HINCS or GMEM/0.5% HINCS, for 24 h and again for 4 h prior to harvest. At harvest, monolayers were rinsed twice with PBS to remove any apoptotic cells in the supernatant, and the monolayers were stripped with trypsin and EDTA as before. Nuclei were prepared by isotonic lysis and centrifugation through a glycerol gradient (Arends *et al.*, 1990). Approximately $10^7$ nuclei were resuspended in 200 μl incubation buffer containing 100 mM Tris-HCl, 2 mM $CaCl_2$, with or without $MgCl_2$ at 2 mM, and incubated at 37°C for 18 h. Previous studies in this laboratory (data not shown) have suggested that these conditions are likely to produce maximal demonstration of endonuclease activity with preference for internucleosomal cleavage. 2 mM zinc ions were also added to the incubation buffer in some experiments to inhibit nuclease activity (Cohen & Duke, 1984). DNA from these incubations was phenol/chloroform extracted, ethanol precipitated and analysed by 2% agarose gel electrophoresis, to reveal chromatin cleavage by the endogenous endonuclease activity. Prior to incubation, more than 99% of the nuclei were found to be non-apoptotic as judged by acridine orange staining and UV fluorescence microscopy.

Chromatin cleavage into the characteristic oligonucleosomal 'ladder' pattern of apoptosis was observed. To obtain a quantitative measure of the extent of digestion to lower order nucleosomal fragments, photographs of the ethidium bromide stained gels were scanned by laser densitometer (Ultrascan XL, enhanced laser densitometer, LKB). For each cell line a 'Digestion Ratio' (DR) was calculated. The DR numerator was the amount of DNA fluorescence in the lower order oligonucleosomes that comprised the mono-and dinucleosomes together with the lower half of the trinucleosomal DNA band (an arbitrary cut off chosen for its accurate reproducibility on the densitometry graph), whereas the DR denominator was the total DNA fluorescence in the electrophoretic track, including the high molecular weight DNA smear.

### Results

### *Myc and ras transfectants show similar measures of proliferation but divergent apoptotic rates*

The six cell lines resulting from transfection of 208F with c-*myc* and T24-*ras* DNA all showed closely similar proliferation rates, analysed as Growth Factions (S + G2/M phases of the cell cycle) during growth in log phase in 10% serum, and there were no statistically significant differences by ANOVA or Kruskal-Wallis tests (Table I). In contrast, the apoptotic indices differed from each other by more than fifteen-fold under these conditions (Figures 1 and 2a). The three c-*myc* transfectants showed significantly higher levels than the control 208F ($P < 0.004$ in call cases), and apoptosis was further increased by serum deprivation (0.01% serum) ($P < 0.004$ in all cases) (Figure 1). Two of the three T24-*ras* transfectants, T1 and T2, showed significantly reduced apoptosis compared to the control 208F ($P = 0.004$ and $P = 0.005$ respectively) (Figure 2a). T3, the third of the T24-*ras* transfectants, showed an apoptotic index not significantly different from the parental line. As expected, these large differences in apoptosis had great effects on the overall population expansion rates of the monolayers as a whole, and these two parameters showed a strong inverse correlation ($r = -0.77$) for the six oncogene transfectants (Table I).

### *High expression of mutated ras is associated with reduced apoptosis*

We sought to establish the role of *ras* in modifying apoptosis by two classes of experiments. In the first, the three *ras* transfected lines were compared in terms of apoptotic rate and relative levels of expression of p21[ras], measured semi-quantitatively as the dilution of antibody Y13-259 at which membrane staining of monolayer-cultured cells was no longer evident. Whereas expression of p21[ras] in T3 was approximately two-fold greater than in the parental line, T2 exhibited four-fold greater expression, and T1 ten-fold greater expression by this method (Figure 2b). These semi-quantitative assessments of p21[ras] protein expression correlated well with estimates of the relative copy number of the transfected genes obtained from Southern hybridisation and densitometric analysis of the three cell lines (data not shown). The levels of p21[ras] detected showed an inverse correlation with apoptotic indices ($r = -0.72$). Growing in log phase in 10% serum, apoptosis was lowest in the cell line with the highest copy number and p21[ras] expression (T1) and highest in T3 in which expression and copy number were lowest. T2 showed an intermediate pattern both in p21[ras] expression and apoptosis. Even in serum-deprived conditions, T1 showed low levels of apoptosis and retained high expression of p21[ras]. In contrast, both T2 and T3 showed elevated apoptotic rates under these conditions with reduction in p21[ras] expression to undetectable levels (data not shown).

**Table I** Cell turnover parameters

| *Cell line* | *Growth fraction* | *Population expansion* | *Apoptotic index* |
|---|---|---|---|
| 208F | 41.7 (± 2.98) | 1.53 | 6.98 (± 1.75) |
| M1 | 44.3 (± 2.17) | 2.89 | 26.5 (± 7.97) |
| M7 | 41.8 (± 3.37) | 1.54 | 30.2 (± 11.1) |
| M8 | 45.5 (± 2.96) | 1.20 | 15.4 (± 4.44) |
| T1 | 46.2 (± 1.17) | 3.56 | 1.7 (± 0.14) |
| T2 | 45.9 (± 1.62) | 3.67 | 2.2 (± 0.12) |
| T3 | 41.0 (± 1.32) | 1.77 | 8.4 (± 3.07) |

Growth Fraction data (S + G2/M phases of the cell cycle analysed flow cytometrically using the PARA 1 program) are means (± s.e.m.) of 9–10 experiments and show no statistically significant differences by ANOVA or Kruskal-Wallis tests. Population Expansion (PE) data are means of triplicate experiments. Apoptotic Index (AI) data (at 10% serum) are means (± s.e.m.) of 6–21 experiments and were log transformed to normalise the distributions for statistical analysis. The means of the log AI values for the six oncogene transfectants correlate inversely ($r = -0.77$) with their PE values.

**Figure 1** The 3 c-*myc* transfectants M1, M7 and M8 show more apoptosis than the control 208F, when grown at high (10%) serum ($P < 0.004$ for all comparisons, Student's *t*-test on log AI data, based on eight experiments). There is a further increase in apoptosis during growth in low (0.01%) serum, compared with the high serum values and that of the control 208F at low serum ($P < 0.004$ in all comparisons, Student's *t*-test on log AI data, based on 20 experiments).

**Figure 2** **a**, The 2 T24-*ras*-transfectants, T1 and T2 show less apoptosis than the control 208F cells, when grown at 10% serum ($P = 0.004$ and $P = 0.005$ respectively). Under conditions of serum deprivation, T1 shows a lower apoptotic index than 208F ($P = 0.006$), whereas T2 and T3 demonstrate higher rates ($P = 0.004$ and $P < 0.00001$ respectively). The addition of 25 μM mevinolin to serum-deprived cultures resulted in a considerable increase in the apoptotic index for all 3 T24-*ras* transfectants compared to 208F ($P < 0.00001$ in all cases) (all comparisons by Student's *t*-test, based on eight experiments). No significant change in the apoptotic index of 208F was observed. **b**, Apoptotic indices for *ras* transfectants and 208F cells grown at 10% serum are compared with relative levels of p21[ras] expression (arbitrary units). High and intermediate levels of p21[ras] expression (T1 and T2) are associated with significantly lower rates of apoptosis than that of the control, whereas a low level of p21[ras] expression (T3) is associated with an apoptotic index similar to 208F. Apoptotic indices correlated inversely ($r = -0.72$) with p21[ras] expression.

In a second series of experiments the isoprenylation inhibitor mevinolin was used in an attempt to inhibit the biological activity of the p21$^{ras}$ protein by blocking its membrane attachment. Mevinolin, applied to serum-deprived cultures at a concentration known to inhibit p21$^{ras}$ function (Hancock *et al.*, 1989; Schafer *et al.*, 1989; De Clue *et al.*, 1991), had little effect on the apoptotic rate of the parental 208F cell line. In contrast, there was a profound increase in apoptotic rates in all three T24-*ras* transfected lines, compared with apoptosis in its absence ($P<0.001$ in all cases) (Figure 2a). The parental line 208F showed no significant increase in apoptosis following treatment with mevinolin indicating that low level endogenous *ras* within this line is presumably not the prime regulator of apoptosis.

*Cellular content of endogenous endonuclease varies in proportion to susceptibility to apoptosis*

Previous experiments have shown that lymphoma cells about to enter apoptosis accumulate an endogenous endonuclease which can be activated by calcium and magnesium ions (Wyllie *et al.*, 1986*b* and 1992). Elsewhere we have described such cells as 'primed' for apoptosis, to distinguish them from cells lacking this component of the effector pathway of apoptosis (Arends & Wyllie, 1991). It was therefore of interest to compare the nuclear content of this calcium/magnesium sensitive endonuclease (or endonuleases) in log phase cultures of the transfected cell lines. Parallel incubations of nuclear preparations from the parental and transfected cell lines revealed several consistent differences. First, incubation of the nuclei from many of the cell lines for several hours frequently produced internucleosomal cleavage of chromatin which was optimum in the presence of calcium plus magnesium ions, but could be inhibited by zinc ions (Figure 3). In every case, more than 99% of the nuclei showed a normal morphology (i.e. without the stigmata of apoptosis) prior to incubation. The three *myc* transfectant lines (M1, M7 and M8) consistently showed the highest nuclear content of the enzyme, as evident by the intensity of ethidium bromide staining of the chromatin ladder from equivalent cell numbers. The high p21$^{ras}$ expressing cell line T1 consistently showed only minimal evidence of internucleosomal cleavage, even after overnight incubation with calcium and magnesium ions. The two T24-*ras* transfectants with lower levels of p21$^{ras}$ expression (T2 and T3) generated fragmented chromatin to a varying degree, which was most conspicuous in cells harvested after several hours incubation in low serum (Figure 4). At high serum concentration there was some cleavage of chromatin, mostly to large oligonucleosomal fragments, but at low serum a greater proportion of digested chromatin appeared as small oligonucleosomes. This was confirmed by densitometric measurement of DNA bands and calculation of 'Digestion Ratios' (DR). Compared with serum supplemented cells, the DR increased in assays of serum-deprived T2 (from 27% to 45%) and T3 (from 53% to 61%), indicating more complete digestion to smaller oligonucleosomal chains by the endonuclease activity present in populations of serum deprived cells. The calcium and magnesium ionic sensitivity of the endonuclease activity was confirmed in T2 and T3 nuclei prepared from cells grown at both serum concentrations. In contrast, T1 showed no significant endonuclease activity at low serum, whereas M8 demonstrated marked nuclease activity at high serum that was further increased by serum deprivation (Figure 4).

**Figure 3** A, Ionic sensitivity of endogenous endonuclease activity within nuclei prepared from viable cells. This shows DNA extracted from nuclei following autodigestion in the presence or absence of magnesium and calcium ions (tracks marked **a** for both ions, tracks marked **b** for calcium ions only). Although the parent 208F shows little chromatin digestion, the c-*myc* transfectants, M1 and M8, show chromatin cleavage at internucleosomal sites which is more marked when both cations are present. Samples from the T24-*ras* transfectant T1 show only high molecular weight DNA with no detectable endonuclease activity. **B**, In a separate experiment M7 shows a similar pattern of chromatin cleavage to M1 and M8. Furthermore, M8 chromatin autodigestion in the presence of magnesium and calcium ions is completely inhibited by zinc ions (track marked **c**). One kb ladder marker tracks (M) are included and these indicate that chromatin is cleaved into fragments that are multiples of the length of DNA wrapped around a single nucleosome (180–200 bp).

### Discussion

*C-*myc *is associated with a high turnover state*

The data show that cell lines of common rat fibroblast parentage can vary fifteen-fold in rates of apoptosis, whilst retaining similar cell proliferation kinetics. The high apoptotic indices in the c-*myc* transfectants confirm the observation made by ourselves and others that constitutive c-*myc* activation is associated with initiation of apoptosis, particularly in cells in which cell-cycle progression is inhibited by a variety of circumstances including reduced serum growth factor support (Wyllie *et al.*, 1987 and 1992; Askew *et al.*, 1991; Bertrand *et al.*, 1991; Evan *et al.*, 1992). However, there is incomplete data regarding the regulation of apoptosis by endogenous *myc* in normal fibroblasts. These conclusions may not apply to all cell types as some appear to die by apoptosis following the disappearance of *myc* (Yuh & Thompson, 1989). The c-*myc* transfectants reported here appear to be in a 'high turnover' state whilst growing in log phase, in which cell proliferation and death by apoptosis co-exist, so that the overall population expansion is substantially slower than expected from consideration of the cell

**Figure 4** Serum sensitivity of endogenous endonuclease activity in viable nuclei. Cells were grown for 24 h and a further 4 h in either low serum (0.05% HINCS - tracks marked Lo) or high serum (10% HINCS - tracks marked Hi) prior to analysis. M8 shows autodigestion of chromatin into oligonucleosomal fragments at both serum concentrations, with an increase in digestion to lower order oligonucleosomes at low serum levels. T1 shows no significant chromatin digestion at either high or low serum. T2 and T3, show patterns of chromatin digestion which differ according to the serum concentration prior to cell harvest and the ionic content of the assay solution. Chromatin digestion in the presence of both calcium and magnesium ions (tracks marked **CM**) is consistently greater than with calcium ions alone (tracks marked **C**). Both T2 and T3, cultured in high serum, show low or intermediate levels of chromatin digestion, the resultant fragments being relatively depleted in low order oligonucleosomes. In contrast, after culture in low serum, chromatin digestion into lower order oligonucleosomes is greater. The overall pattern is one of serum sensitive expression of endonuclease activity in T2 and T3. One kb ladder marker track (**M**) is included.

proliferation rate alone. There was no other obvious means of exit from the proliferating pool in these cultures (such as necrosis or differentiation). Thus, the rate of apoptosis appears to be the major regulator of the rate of population expansion for these cell lines growing in culture. Furthermore, high turnover states of this sort are almost universal in tumours growing *in vivo* in which deregulation of c-*myc* is also a common event (Field & Spandidos, 1990), supporting an important role for apoptosis in determining net tumour growth *in vivo*, although c-*myc* may not be the only cellular protein of significance in this regard.

*Mutated Ha-*ras *is associated with suppression of apoptosis*

Of the mutated Ha-*ras* transfectants, two lines showed suppressed apoptosis relative to the control. In experiments of this design, in which *ras* expression is not selectively reversible, it is not possible to be completely certain that the differences in apoptotic rate are due exclusively to expression of the transfected oncogene rather than incidental cellular changes arising during transfection and selection. We show here, however, that inhibition of apoptosis is proportional to expression of functional p21$^{ras}$ protein in the three independent transfectants, following serum withdrawal and immediately after blockade of p21$^{ras}$ processing by mevinolin in dosages previously shown to be effective in inhibition of isoprenylation (Hancock *et al.*, 1989; Schafer *et al.*, 1989; DeClue *et al.*, 1991). Strikingly, this pharmacological blockade induced similarly high apoptotic rates in all the three T24-*ras* transfectants although there was no immediate toxic effect in the parental 208F fibroblast. Inhibition of *ras* expression has been reported to precede, and perhaps be a prerequisite for apoptosis of cultured chloroleukemic cells (Servomaa & Rytomaa, 1988). In a mouse mast cell line transfected with human activated Ha-*ras* oncogene in a regulable construct, *ras* activation was also associated with rapid growth, whilst reduced *ras* expression was accompanied by cell death (Andrejauskas & Moroni, 1989).

*Susceptibility to apoptosis reflects availability of endonuclease in viable cells*

We have argued elsewhere that entry to apoptosis requires two cellular events: 'priming', in which the effector proteins for apoptosis accumulate within the cell, and 'triggering', in which these proteins are activated (Arends & Wyllie, 1991; Dive & Wyllie, 1993). By implication, cells might exist in an unprimed state in which apoptosis would be impossible, at least temporarily, until the effector proteins accumulated. Certain cell types such as cortical thymocytes appear sensitive to apoptosis induced by a wide variety of disparate agents: ligands for the T cell receptor, ionising radiation, etoposide, toxic agents such as TCD-Dioxin, or steroid hormones (Van Haelst, 1967; Umansky *et al.*, 1981; Wyllie & Morris, 1982; Wyllie *et al.*, 1984; McConkey *et al.*, 1988; Yamada & Ohyama, 1988; Smith *et al.*, 1989; Walker *et al.*, 1991). These stimuli have clearly different initial effects upon the thymocyte, as shown by the fact that calcium signalling (McConkey *et al.*, 1989*a* and 1989*b*) and p53 dependence (Clarke *et al.*, 1993) are features of some but not all of them. Nonetheless, apoptosis is the final common event, complete with chromatin condensation and activation of an endogenous endonuclease which appears to be present constitutively in these cells (Wyllie, 1980; Arends *et al.*, 1990). Similarly, divergent stimuli such as c-*myc* expression together with growth arrest or exposure to etoposide can induce apoptosis in cultured fibroblasts (Evan *et al.*, 1992; Fanidi *et al.*, 1992). However, fibroblasts and lymphocytes can be rendered insensitive to apoptosis in response to a similar variety of stimuli by suitable gene expression, such as *ras* as shown here and *bcl*-2 (Vaux *et al.*, 1988; Nunez *et al.*, 1990; Hockenbery *et al.*, 1990; Evan *et al.*, 1992; Fanidi *et al.*, 1992; Bissonnette *et al.*, 1992; Wyllie *et al.*, 1992). The mechanism whereby these changes in susceptibility to apoptosis are engendered is not known, but one of the possibilities is that elements within the apoptosis effector pathway may themselves be regulable.

This proposition is difficult to test as no element of the effector pathway has been definitively identified and purified. In this paper, we have examined the availability of a nuclear calcium/magnesium sensitive endonuclease (or endonucleases), whose activity is apparently responsible for the chromatin cleavage of apoptosis and is induced prior to the onset of chromatin condensation (Wyllie *et al.*, 1986*a*, 1986*b*, 1992 and 1993; Arends *et al.*, 1990; Walker *et al.*, 1991). Endonuclease activation appears to be a mid to late event in apoptosis, as in programmed cell death in other circumstances (Hengartner *et al.*, 1992) and may provide a useful indication of the cells' ability to undergo the process as a whole. Here we demonstrate that abundance of readily activated endonuclease in viable fibroblast nuclei is associated with susceptibility of these cells to undergo apoptosis, being high in c-*myc* transfectants, apparently absent in the T24-*ras* transfectant, T1, with the lowest apoptotic rate and the highest expression of p21$^{ras}$ oncoprotein, and substantially increased in serum-deprived cultures of the intermediate and low p21$^{ras}$ expressing lines, T2 and T3, in which apoptosis is also increased. Therefore, these experiments provide evidence for a mechanism whereby oncogenes may regulate apoptosis, even in the absence of definitive information concerning any of the effector molecules. Hopefully, when these effectors are isolated, it will be possible to test more precisely the hypothesis advanced here.

*The role of oncogene modulation of apoptosis in tumour growth*

Finally, the question arises as to the role of *ras* and *myc* oncogenes in the growth of authentic human tumours. The data reported here show that differences in *myc* and *ras* oncogene expression, are not limited to effects upon cell proliferation, but can profoundly influence overall population expansion through modifying apoptosis. Elsewhere we show (Arends *et al.* unpublished data) that these transfected cell lines have differences in their rates and aggressiveness of tumour growth *in vivo* that correspond to the phenomena described here *in vitro*, extending the previously published data (Wyllie *et al.*, 1987) that showed that a T24-*ras* expressing cell line (T1) produced a higher primary tumour take-rate and a significantly higher proportion of test mice with metastasis at 14 days, compared with a c-*myc* expressing line (M1). There is also evidence from human pathology that expression of *myc* is associated with tumours with high cell turnover (Field & Spandidos, 1990), whereas *ras* expression or oncogenic activation is associated with cell population expansion in potentially premalignant tumours of the colon and breast, rather than acquisition of specific features of the malignant phenotype (Williams *et al.*, 1985; Going *et al.*, 1992). Thus, the data support the hypothesis that oncogenes such as *ras* and *bcl*-2 can suppress apoptosis (Vaux *et al.*, 1988; Hockenbery *et al.*, 1990; Nunez *et al.*, 1990). Reduction of apoptosis directly causes an increase in population expansion likely to produce greater retention of cells acquiring new mutations (Wyllie, 1985), increasing the risk of malignant transformation in a population of cells in a premalignant lesion, or increasing tumour progression in an already malignant population of cells, and both of these have been associated with *ras* activation (Brown *et al.*, 1986; Buchmann *et al.*, 1991). Furthermore, genetic modulation of apoptosis, by *myc*, *bcl*-2 and p53, determine to a large extent the cellular response to certain anticancer chemotherapeutic agents such as etoposide (Walker *et al.*, 1991; Fanidi *et al.*, 1992; Clarke *et al.*, 1993; Evans & Dive, 1993). Thus, the levels of apoptosis in tumours appear to influence net growth, acquisition of new properties, and response to certain cytotoxic agents, and susceptibility to apoptosis is regulated by many genes including *myc* and *ras*.

M.J.A. was a Medical Research Council Training Fellow; the authors also thank the Cancer Research Campaign, Scottish Home and Health Department, and Sir Stanley and Lady Davidson Research Fund for financial support.

### References

ANDREJAUSKAS, E. & MORONI, C. (1989). Reversible abrogation of IL-3 dependence by an inducible H-*ras* oncogene. *EMBO J.*, **8**, 2575–2581.

ARENDS, M.J., MORRIS, R.G. & WYLLIE, A.H. (1990). Apoptosis: the role of the endonuclease. *Am. J. Pathol.*, **136**, 593–608.

ARENDS, M.J. & WYLLIE, A.H. (1991). Apoptosis: mechanisms and roles in pathology. *Int. Rev. Exp. Pathol.*, **32**, 223–254.

ASKEW, D., ASHMUN, R., SIMMONS, B. & CLEVELAND, J. (1991). Constitutive c-*myc* expression in IL-3 dependent myeloid cell line suppresses cycle arrest and accelerates apoptosis. *Oncogene*, **6**, 1915–1922.

BERTRAND, R., SARANG, M., JENKIN, J., KERRIGAN, D. & POMMIER, Y. (1991). Differential induction of secondary DNA fragmentation by topoisomerase II inhibitors in human tumor cells lines with amplified c-*myc* expression. *Cancer Res.*, **51**, 6280–6285.

BISSONNETTE, R.P., ECHEVERRI, F., MAHBOUBI, A. & GREEN, D.R. (1992). Apoptotic cell death induced by c-*myc* is inhibited by *bcl*-2. *Nature*, **359**, 552–554.

BOS, J.L., FEARON, E.R., HAMILTON, S.R. & 4 others (1987). Prevalence of *ras* gene mutations in human colorectal cancers. *Nature*, **327**, 293–297.

BROWN, K., QUINTANILLA, M., RAMSDEN, M., KERR, I.B., YONG, S. & BALMAIN, A. (1986). V-*ras* genes from Harvey and BALB murine sarcoma viruses can act as initiators of two-stage mouse skin carcinogenesis. *Cell*, **46**, 447–456.

BUCHMANN, A., RUGGERI, B., KLEIN-SZANTO, A.J.P. & BALMAIN, A. (1991). Progression of squamous carcinoma cells to spindle carcinomas of mouse skin is associated with an imbalance of H-*ras* alleles on chromosome 7. *Cancer Res.*, **57**, 4097–4101.

CLARKE, A.R., MAANDAG, E.R., VAN ROON, M., VAN DER LUGT, N.M., VAN DER VALK, M., HOOPER, M.L., BERNS, A. & TE RIELE, H. (1992). Requirement for a functional Rb-1 gene in murine development. *Nature*, **359**, 328–330.

CLARKE, A.R., PURDIE, C.A., HARRISON, D.J., MORRIS, R.G., BIRD, C.C., HOOPER, M.L. & WYLLIE, A.H. (1993). Thymocyte apoptosis induced by p53 – dependent and independent pathways. *Nature*, **362**, 849–852.

COHEN, J.J. & DUKE, R.C. (1984). Glucocorticoid activation of a calcium dependent endonuclease in thymocyte nuclei leads to cell death. *J. Immunol.*, **132**, 38–42.

DECLUE, J.E., VASS, W.C., PAPAGEORGE, A.G., LOWY, D.R. & WILLUMSEN, B.M. (1991). Inhibition of cell growth by lovastatin is independent of *ras* function. *Cancer Res.*, **51**, 712–717.

DIVE, C. & WYLLIE, A.H. (1993). Apoptosis and cancer chemotherapy. In *Frontiers in Pharmacology: Cancer Chemotherapy*. Hickman, J.A. & Tritton, T.T. (eds), Blackwell Scientific: Oxford pp. 21–56.

EVANS, C.A., OWEN, P.J., WHETTON, A.D. & DIVE, C. (1993). Activation of the Abelson tyrosine kinase activity is associated with the suppression of apoptosis in haemopoietic cells. *Cancer Res.*, **53**, 1735–1738.

EVANS, D.L. & DIVE, C. (1993). Effects of cisplatin on the induction of apoptosis in proliferating hepatoma cells and non-proliferating immature thymocytes. *Cancer Res.*, **53**, 2133–2139.

EVAN, G.I., WYLLIE, A.H., GILBERT, C.S. & 6 others (1992). Induction of apoptosis in fibroblasts by c-*myc* protein. *Cell*, **69**, 119–128.

FANIDI, A., HARRINGTON, E.A. & EVAN, G.I. (1992). Interaction between c-*myc* and *bcl*-2 proto-oncogenes: a novel paradigm for oncogene cooperation. *Nature*, **359**, 554–556.

FIELD, J.K. & SPANDIDOS, D.A. (1990). The role of *ras* and *myc* oncogenes in human solid tumours and their relevance in diagnosis and prognosis. *Anti Cancer Res.*, **10**, 1–22.

FURTH, M.E., DAVIS, L.J., FLEURDELYS, B. & SCOLNICK, E.M. (1982). Monoclonal antibodies to the p21 products of the transforming gene of Harvey murine sarcoma virus and of the cellular *ras* gene family. *J. Virol.*, **43**, 294–304.

GOING, J.J., WILLIAMS, A.R.W., WYLLIE, A.H., ANDERSON, T.J. & PIRIS, J. (1988). Optimal preservation of p21 *ras* immunoreactivity and morphology in paraffin-embedded tissue. *J. Pathol.*, **155**, 185–190.

GOING, J.J., ANDERSON, T.J. & WYLLIE, A.H. (1992). *Ras* p21 in breast tissue: associations with pathology and cellular localisation. *Br. J. Cancer*, **65**, 45–50.

HANCOCK, J.F., MAGEE, A.I., CHILDS, J.E. & MARSHALL, C.J. (1989). All *ras* proteins are polyisoprenylated but only some are palmitoylated. *Cell*, **57**, 1167–1177.

HENGARTNER, M.O., ELLIS, R.E. & HORVITZ, H.R. (1992). Caenorhabditis elegans gene ced-9 protects cells from programmed cell death. *Nature*, **356**, 494–499.

HOCKENBERY, D., NUNEZ, G., MILLIMAN, C., SCHREIBER, R.D. & KORSMEYER, S.J. (1990). *Bcl*-2 is an inner mitochondrial membrane protein that blocks programmed cell death. *Nature*, **348**, 334–336.

JACKS, T., FAZELI, A., SCHMITT, E.M., BRONSON, R.T., GOODELL, M.A. & WEINBERG, R.A. (1992). Effects of an Rb mutation in the mouse. *Nature*, **359**, 295–300.

KERR, J.F.R., WYLLIE, A.H. & CURRIE, A.R. (1972). Apoptosis: a basic biological phenomenon with wide-ranging implications in tissue kinetics. *Br. J. Cancer*, **26**, 239–257.

KITA, T., BROWN, M.S. & GOLDSTEIN, J.L. (1980). Feedback regulation of 3-hydroxy-3-methylglutaryl coenzyme A reductase in livers of mice treated with mevinolin, a competitive inhibitor of the reductase. *J. Clin. Invest.*, **66**, 1094–1100.

LEE, E.Y.-H.P., CHANG, C.-Y., HU, N., WANG, Y.-C.J., LAI, C.-C., HERRUP, K. & LEE, W.-H. (1992). Mice deficient for Rb are nonviable and show defects in neurogenesis and haematopoiesis. *Nature*, **259**, 288–294.

McCONKEY, D.J., HARTZELL, P., DUDDY, S.K., HAKANSSON, H. & ORRENIUS, S. (1988). 2, 3, 7, 8 – Tetrachlorodibenzo-p-toxin kills immature thymocytes by $Ca^{2+}$-mediated endonuclease activation. *Science*, **242**, 256–259.

McCONKEY, D.J., NICOTERA, P., HARTZELL, P., BOLLOMA, G., WYLLIE, A.H. & ORRENIUS, S. (1989*a*). Glucocorticoids activate a suicide process in thymocytes through an elevation of cytosolic $Ca^{2+}$ concentration. *Arch. Biochem. Biophys.*, **269**, 365–370.

McCONKEY, D.J., HARTZELL, P., AMADOR-PEREZ, J.F., ORRENIUS, S. & JONDAL, M.J. (1989*b*). Calcium-dependent killing of immature thymocytes by stimulation via the CD3/T cell receptor complex. *J. Immunol.*, **143**, 1801–1806.

NUNEZ, G., LONDON, L., HOCKENBERY, D., ALEXANDER, M., McKEARN, J.P. & KORSMEYER, S.J. (1990). Deregulated *bcl*-2 gene expression selectively prolongs survival of growth factor-deprived haemopoietic cell lines. *J. Immunol.*, **144**, 3602–3610.

PEROTTI, M., TODDEI, F., MIRABELLI, F. & 4 others (1990). Calcium-dependent DNA fragmentation in human synovial cells exposed to cold shock. *FEBS Lett.*, **259**, 331–334.

QUADE, K. (1979). Transformation of mammalian cells by avian myelocytomatosis virus and avian erythroblastosis virus. *Virology*, **98**, 461–465.

SANTOS, E., TRONICK, S.R., AARONSON, S.A., PULCIANI, S. & BARBACID, M. (1982). T24 human bladder carcinoma oncogene is an activated form of the normal human homologue of BALB- and Harvey-MSV transforming genes. *Nature*, **298**, 343–347.

SCHAFER, W.R., KIM, R., STERNE, R., THORNER, J., KIM, S. & RINE, J. (1989). Genetic and pharmacological suppression of oncogenic mutations in *ras* genes of yeast and humans. *Science*, **245**, 379–385.

SERVOMAA, K. & RYTOMAA, T. (1988). Suicidal death of rat chloroleukaemia cells by activation of the long interspersed repetitive DNA element (L1Rn). *Cell Tissue Kinet.*, **21**, 33–43.

SHAW, P., BOVEY, R., TARDY, S., SAHLI, R., SORDAT, B. & COSTA, J. (1992). Induction of apoptosis by wild-type p53 in a human colon tumor-derived cell line. *Proc. Natl Acad. Sci. USA*, **89**, 4495–4499.

SMITH, C.A., WILLIAMS, G.T., KINGSTON, R., JENKINSON, E.J. & OWEN, J.J.T. (1989). Antibodies to CD3/T-cell receptor complex induce death by apoptosis in immature T-cells in thymic cultures. *Nature*, **337**, 181–184.

SPANDIDOS, D.A. & WILKIE, N.M. (1984). Malignant transformation of early passage rodent cells by a single mutated human oncogene. *Nature*, **310**, 469–475.

UMANSKY, S.R., KOROL, B.A. & NELIPOVICH, P.A. (1981). *In vivo* DNA degradation in thymocytes of x-irradiated or hydrocortisone-treated rats. *Biochim. Biophys. Acta*, **655**, 9–17.

VAN HAELST, U. (1967). Light and electron microscopic study of the normal and pathological thymus of the rat. II. The acute thymic involution. *Z. Zellforsch. Mikrosk. Anat.*, **80**, 153–182.

VAUX, D.L., CORY, S. & ADAMS, J.M. (1988). *Bcl*-2 gene promotes haemopoietic cell survival and cooperates with c-*myc* to immortalize pre-B cells. *Nature*, **335**, 440–442.

VINDELOV, L.L., CHRISTENSEN, I.J. & NISSEN, N. (1983). Standardization of high-resolution flow cytometric DNA analysis by the simultaneous use of chicken and trout red blood cells as internal reference standards. *Cytometry*, **3**, 328–331.

WALKER, P.R., SMITH, C., YOUDALE, T., WHITFIELD, J.F. & SOKORSKA, M. (1991). Topoisomerase II – reactive chemotherapeutic drugs induce apoptosis in thymocytes. *Cancer Res.*, **51**, 1078–1085.

WILLIAMS, A.R.W., PIRIS, J., SPANDIDOS, D.A. & WYLLIE, A.H. (1985). Immunohistochemical detection of the *ras* oncogene p21 product in an experimental tumour and in human colorectal neoplasms. *Br. J. Cancer*, **52**, 687–693.

WYLLIE, A.H. (1980). Glucocorticoid induced thymocyte apoptosis is associated with endogenous endonuclease activation. *Nature*, **284**, 555–556.

WYLLIE, A.H., KERR, J.F.R. & CURRIE, A.R. (1980). Cell death: The significance of apoptosis. *Int. Rev. Cytol.*, **68**, 251–306.

WYLLIE, A.H. & MORRIS, R.G. (1982). Hormone-induced cell death, purification and properties of thymocytes undergoing apoptosis after glucocorticoid treatment. *Am. J. Pathol.*, **109**, 78–87.

WYLLIE, A.H., MORRIS, R.G., SMITH, A.L. & DUNLOP, D. (1984). Chromatin cleavage in apoptosis: association with condensed chromatin morphology and dependence on macromolecular synthesis. *J. Pathol.*, **142**, 67–77.

WYLLIE, A.H. (1985). The biology of cell death in tumours. *Anticancer Res.*, **5**, 131–136.

WYLLIE, A.H., MORRIS, R.G., ARENDS, M.J. & WATT, A.E. (1986*a*). Nuclease activation in programmed cell death. In: *Coordinated regulation of gene expression*. Clayton, R.M. & Truman, D.E.S. (eds), Plenum Press: New York. pp. 33–41.

WYLLIE, A.H., MORRIS, R.G. & WATT, A.E. (1986*b*). Terminally differentiated and dying lymphoid cells contain nuclear endonuclease potentially responsible for chromatin changes of apoptosis. *J. Pathol.*, **148**, 94A.

WYLLIE, A.H. (1987). Cell death. *Int. Rev. Cytol. (Suppl)*, **17**, 755–785.

WYLLIE, A.H., ROSE, K.A., MORRIS, R.G., STEEL, C.M., FOSTER, E. & SPANDIDOS, D.A. (1987). Rodent fibroblast tumours expressing human *myc* and *ras* genes: growth, metastasis and endogenous oncogene expression. *Br. J. Cancer*, **56**, 251–259.

WYLLIE, A.H., ARENDS, M.J., MORRIS, R.G., WALKER, S.W. & EVAN, G. (1992). The apoptosis endonuclease and its regulation. *Seminars in Immunol.*, **4**, 389–397.

WYLLIE, A.H., ARENDS, M.J., HOGG, R.M. & NUNN, A. (1993). DNA degradation – double-strand breaks. *In Vitro Toxicity Indicators (Methods in Toxicology)*. (in press).

YAMADA, T. & OHYAMA, H. (1988). Radiation-induced interphase death of rat thymocytes is internally programmed (apoptosis). *Int. J. Radiat. Biol.*, **53**, 65–75.

YONISH-ROUACH, E., RESNITZKY, D., LOTEM, J., SACHS, L., KIMCHI, A. & OREN, M. (1991). Wild type p53 induces apoptosis of myeloid leukaemic cells that is inhibited by interleukin-6. *Nature*, **353**, 345–347.

YUH, Y.-S. & THOMPSON, E.B. (1989). Glucocorticoid effect on oncogene/growth gene expression in human T lymphoblastic leukemic cell line CCRF-CEM. *J. Biol. Chem.*, **264**, 10904–10910.